# Integration of Hot Isostatic Pressing and Heat Treatment for Advanced Modified γ-TiAl TNM Alloys

**DOI:** 10.3390/ma15124211

**Published:** 2022-06-14

**Authors:** Daniel Bernal, Xabier Chamorro, Iñaki Hurtado, Inmaculada Lopez-Galilea, David Bürger, Sebastian Weber, Iñaki Madariaga

**Affiliations:** 1 Mechanical and Manufacturing Department, Mondragon University, Loramendi 4, 20500 Arrasate-Mondragón, Spain; xchamorro@mondragon.edu (X.C.); ihurtado@mondragon.edu (I.H.); 2 Institute for Materials, Ruhr University Bochum, Universitätsstr. 150, 44801 Bochum, Germany; lopez@wtech.rub.de (I.L.-G.); david.buerger@ruhr-uni-bochum.de (D.B.); weber@wtech.rub.de (S.W.); 3 Materials and Processes Department, Industria de Turbopropulsores, SAU, Parque Tecnológico No 300, 48170 Zamudio, Spain; inaki.madariaga@itpaero.com

**Keywords:** titanium aluminides, integrated hot isostatic pressing heat treatment, creep, investment casting, chemical composition

## Abstract

The conventional processing route of TNM (Ti-Nb-Mo) alloys combines casting and Hot Isostatic Pressing (HIP) followed by forging and multiple heat treatments to establish optimum properties. This is a time-consuming and costly process. In this study we present an advanced alternative TNM alloy processing route combining HIP and heat treatments into a single process, which we refer to as IHT (integrated HIP heat treatment), applied to a modified TNM alloy with 1.5B. A Quintus HIP lab unit with a quenching module was used, achieving fast and controlled cooling, which differs from the slow cooling rates of conventional HIP units. A Ti-42.5Al-3.5Nb-1Mo-1.5B (at.%) was subjected to an integrated two HIP steps at 200 MPa, one at 1250 °C for 3 h and another at 1260 °C for 1 h, both under a protective Ar atmosphere and followed by cooling at 30 K/min down to room temperature. The results were compared against the Ti-43.5Al-3.5Nb-1Mo-0.8B (at.%) thermomechanically processed in a conventional way. Applying IHT processing to the 1.5B alloy does indeed achieve good creep strength, and the secondary creep rate of the IHT processed materials is similar to that of conventionally forged TNM alloys. Thus, the proposed advanced IHT processing route could manufacture more cost-effective TiAl components.

## 1. Introduction

Intermetallic γ-TiAl based alloys are attracting considerable attention as emerging lightweight materials because they combine high specific strength with strong resistance to oxidation and corrosion according to [1,2]. As a result, [1] pointed out that these materials are suitable for aircraft engines and gas-burning power-generation plants. The present work focusses on β-solidifying γ-TiAl TNM (Ti-Nb-Mo) alloys, which are used to manufacture turbine blades for low pressure components of geared turbofans as stated by [3], allowing a reduction in fuel consumption, pollutants and noise emissions, as well as operating costs according to [4].

A series of processes are required to manufacture conventional TNM alloys, which involve casting, forging, HIP, and multiple-step heat treatments (HT). The conventional route starts with two-step Vacuum Arc Remelting (VAR) melting which achieves chemical homogeneity. Ref. [5,6] showed that the homogenized electrodes are melted in a VAR Skull Melter and cast into billets via spin casting into permanent moulds. These ingots are then subjected to HIP at 1200 °C for 4 h at 200 MPa in Ar atmosphere and subsequently subjected to a hot-die forging step. Finally, the material undergoes a three-step heat treatment (HT) to achieve a homogeneous microstructure. The first step consists of a long-term homogenization annealing of several hours at high temperature, with subsequent air-cooling. This is followed by a second short-term high-temperature annealing above 1250 °C in the (α + β + γ) phase field, with a subsequent air/furnace cooling. In the final step, stabilization annealing is performed at 900 °C/6 h followed by air-cooling.

The processes described above affect microstructural evolution. HIP is a necessary step to reduce the number of voids and micropores in the material. In the case of the TNM alloys, HIP is typically performed by heating the material up to 1200 °C, holding it for 4 h under 200 MPa, and cooling it (cooling rate: <8 K/min) to room temperature. According to [6], during this process a decomposition reaction within the α_2_/γ-colonies in accordance with α_2_ → β_0_ + γ takes place, which leads to an increase in thickness in the γ-lamellae, as well as the formation of secondary precipitates in the β_0_-phase (referred to as β_0,sec_) within the α_2_/γ-colonies. In addition, ref. [7] pointed out that detrimental ω-domains can be found in the β_0_-phase. Thus, further processing is required to achieve the required mechanical properties after reducing the internal porosity with HIP.

**Figure 1:**
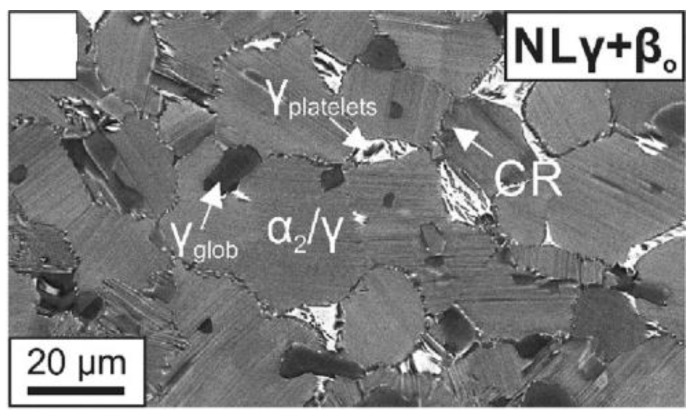
BSE image showing microstructure TNM-Forged-NLGB. Reprinted with permission from Ref. [6]. 2018, M. Kastenhuber et al.

The microstructure obtained from the conventional three-step HT processing route features fine-grained lamellar α_2_/γ-colonies with an average grain size of 25 µm and a lamellar spacing of 22 nm, including a 29 wt. % α_2_, 5 wt. % β_0_, and 66 wt. % γ, analysed by [6]. We refer to this microstructure as TNM-Forged-NLGB (Nearly Lamellar Globular γ on the colony boundaries), and it is the reference microstructure used in the present work. A scanning electron microscope (SEM) micrograph is presented in Figure 1.

**Figure 2:**
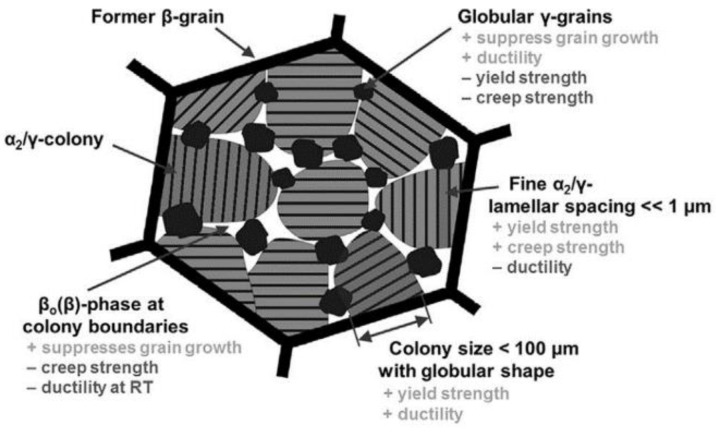
Influence of microstructural constituents on mechanical properties. Reprinted with permission from Ref. [13]. 2017, S. Mayer et al.

The constituents of TNM-Forged-NLGB play a critical role (Figure 2). As [8,9] stated, the β-phase exhibiting a higher number of microscopic crystallographic slip systems, resulting in being softer than the γ-phase, which in turn is softer than the α-phase. In addition, the size and shape of each microstructural constituent have an impact on the mechanical properties. The β-phase suppresses grain growth, reduces creep strength, and diminishes ductility at room temperature. Globular γ-grains suppress grain growth and improve ductility but reduce yield strength and creep resistance. A nanoscale α_2_/γ-lamellar spacing increases yield and creep strength but reduces ductility. From the work of [10,11,12,13], it can be concluded that a lamellar colony size below 100 µm with globular shape enhances yield strength and ductility. Thus, an optimum compromise needs to be found between the conflicting effects of the individual microstructural features. Regarding boron, it plays an important role in TNM alloys, since different types of borides can be precipitated depending on the chemical composition and the solidification cooling rate. This research studies boron contents higher than the critical 0.5 at.% and solidification cooling rates lower than the critical 10 K/s. Thus, the precipitated boride is the blocky one, resulting in a uniformly distribution of borides that enhance microstructural homogeneity.

In the last decade, [5,14] stated that optimum microstructures are typically adjusted by hot-forming operations and further HTs starting from the cast/HIP state. According to [13], grain refinement can also be achieved by a combination of forging and a subsequent recrystallization HT, where recrystallization is accompanied by phase transformations, which affect forging texture and segregation. This latter processing route is tedious and time-consuming. However, several authors [14,15] have argued the need for replacing the complex hot forging process path with specific modified post-HIP treatments, without downgrading mechanical properties. Nevertheless, no attempts have been made to date to establish cost beneficial procedures, which obtain materials with suitable microstructures and mechanical properties directly through integrated HIP/HT-treatments.

Greater efficiency at lower costs can be achieved using the integrated HIP heat treatment (IHT) which reduces the number of processing steps. Modern HIP units can adjust targeted cooling rates, which permit the establishment of desired microstructures during cooling, without the need to perform subsequent thermal treatments. Previous studies from [16,17,18,19] have proven that this new heat treatment process has shown great potential in other metallic systems.

As stated by [20], creep is a time-dependent plastic deformation which occurs under a constant applied load at elevated temperatures. Since TiAl alloys operate under creep conditions, described by [21], creep tests are used to evaluate their performance. Creep curves of TNM alloys exhibit three different stages: Primary creep, where the creep rate decreases with time, results in an instantaneous plastic strain that occurs directly after loading, and is followed by a transient creep where the strain rate decreases with time and strain, until a minimum creep rate is established. At this point, the material enters the secondary creep stage, where the creep rate remains constant and the material spends most of its creep life. During this period, the lamellar structure of the TiAl alloys evolves into a spheroidal microstructure, governed by Dynamic Recrystallization (DRX) processes. The local deformation causes the metastable α_2_ to partially dissolve (predominantly from the α_2_/γ interface) to form the γ-phase. Finally, tertiary creep manifests itself in a rapid increase of creep rate. Tertiary creep is associated with a progressive increase of dislocation density accompanied by ongoing DRX. Ref. [22] revealed that the main factor that accelerates creep rate in TiAl alloys is microstructure degradation related to phase transformation and DRX.

Ref. [6] studied the impact of microstructure on the creep strength of a TNM alloy at 750 °C and 250 MPa, which serves as a reference for the present study. A nearly lamellar microstructure with β_0_-phase and globular γ-phase (referred to as NL_γ+β_0__) exhibited the highest minimum creep rate (ε_min_) of 4.8 × 10^−8^ s^−1^. This was related to a β_0_-phase that facilitated diffusional processes at elevated temperatures and reduced the sliding resistance of the grain boundaries, owing to the less densely packed BCC crystal structure. In contrast, a nearly lamellar microstructure with no β_0_ enhanced the creep strength, by reducing the minimum creep rate to 3.1 × 10^−8^ s^−1^. Finally, a fully lamellar microstructure provided the lowest minimum creep rate (6.6 × 10^−9^ s^−1^) and increased component life because of the elimination of the globular γ-phase that promotes dislocation motion, while borides reduce dislocations motion by pinning microconstituents.

The present work seeks to develop and understand the effects of different thermomechanical processing routes in conjunction with the effect of similar chemical compositions, for cast modified TNM alloys on their microstructures and creep strengths, by integrating HIP and HT into a single process. Recent studies from [16,17,18,19] have shown the benefits of the IHT process, resulting in components with enhanced creep and fatigue resistance.

In this study, we present an advanced alternative manufacturing route for TiAl components that minimizes processing time compared to conventional forged TNM alloys while achieving similar mechanical properties for modified TNM alloys. This IHT processing route is presented together with microstructural analysis and creep testing, to monitor microstructural evolution and assess mechanical performance.

## 2. Materials and Methods

### 2.1. Alloy Manufacturing and Application of Thermomechanical Processes

In this investigation, two TNM-based alloys were cast and subjected to different thermomechanical processes to analyse the effect of chemical composition and thermomechanical history on creep strength. The chemical composition of the two manufactured TNM-based alloys is summarized in Table 1.

The alloys TNM-0.8B-HIP and TNM-0.8B-HIP-HT were manufactured in two ways. First, all samples were melted using a plasma arc furnace to produce Plasma Arc Melting (PAM) ingots of dimensions 60 mm in diameter and 250 mm in length that were subsequently re-melted using an Induction Skull Melting (ISM) unit. All ingots were then melted again in an ISM unit under vacuum and poured into an yttrium-based investment casting mould. The mould was heated to 600 °C and rotated during pouring to create a centrifugal force, which helped fill the mould cavity with the metal and prevent misruns or severe shrinkage. Finally, all samples were subjected to a slow cooling conventional HIP at 200 MPa in Ar atmosphere and 1270 °C for 4 h to reduce the microporosity arising from the casting process, corresponding to the TNM-0.8B-HIP condition. Then, part of the samples, TNM-0.8B-HIP-HT, were subjected to the selected HT at 1260 °C for 1 h with controlled cooling of 30 K/min to room temperature employing the Quintus-QIH9^®^ HIP lab unit, in an Ar atmosphere at 20 MPa, as [23] described.

**Figure 3:**
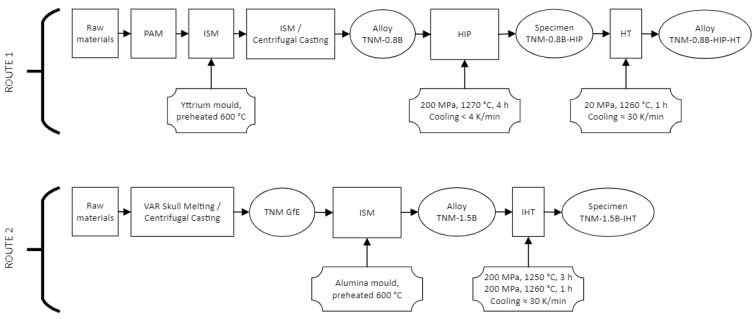
Flow chart of the manufacturing and processing routes.

The novel modified TNM-1.5B-IHT alloy was manufactured by casting plus an integrated HIP/HT cycle, as follows: First, the billets of TNM master alloy of dimensions 57 mm in diameter and 80 mm in length, shipped from GfE, were manufactured by melting the feedstock materials, combining VAR Skull Melting with centrifugal casting in permanent moulds as [24] described, which generated a homogeneous and fine-grained microstructure. Then, the billets of TNM master alloy were melted in an ISM furnace and cast into an alumina ceramic mould at a controlled cooling rate of around 10 K/s. This achieved a homogeneous microstructure with the precipitation of the beneficial blocky boride, as described in reference [25]. Afterwards, the as-cast sample was subjected to an IHT that integrates HIP and HT, allowing an accelerated thermomechanical processing of the alloy, to obtain the final part. This was achieved by HIP at 1250 °C for 3 h at 200 MPa in Ar atmosphere, followed by a HT at 1260 °C for 1 h at 200 MPa in Ar atmosphere. Finally, the part was finished with a cooling rate of 30 K/min to room temperature, as described in reference [23]. The processing routes of all samples are summarised in Table 2 and depicted on Figure 3.

### 2.2. Insights into the IHT Process

**Figure 4:**
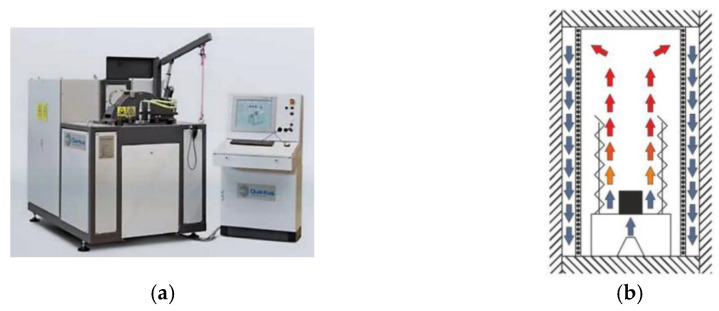
HIP; (**a**) Quintus-QIH9 model and (**b**) URQ^®^ technology.

A Quintus-QIH9^®^ HIP lab unit from the Ruhr-Universität Bochum was used to perform the IHT heat treatment. This model is equipped with a new Uniform Rapid Quenching furnace (URQ^®^) which enables fast and precise cooling from high to low temperatures, as opposed to conventional HIP units, whose cooling is much slower. The QIH9 fast cooling is achieved through heat exchange between the hot gas of the internal chamber and the cold gas outside of the heatshield that is pressed into the hot zone. The hot gas is cooled when passing the heatshield, which acts as a heatsink. At the same time, there is a continuous circulation of gas inside the pressure vessel. This gradient of temperature between the inside and outside gas of the heatshield ensures the fast cooling of the sample (Figure 4).

**Figure 5:**
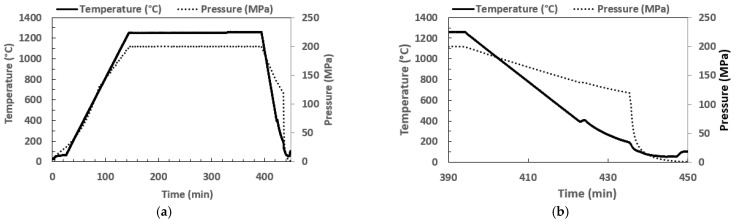
T-t-P profile; (**a**) General, and (**b**) Detail.

Figure 5 plots the temperature-time-pressure profile (T-t-P) for the sample TNM-1.5B-IHT, in which a precise and fast cooling rate was obtained, due to the quenching module of the IHT unit.

### 2.3. Creep and Mechanical Testing

The creep experiments were performed at the Ruhr-Universität Bochum using a miniature tensile creep specimen as described by [26,27]. The specimens were machined with wire spark erosion. Creep machines from Denison-Mayes of type TC 20 Mark II equipped with a three-zone resistance furnace were used. During creep testing the miniature flat specimens with an initial gauge length of 22 mm and an initial width of 2 mm were positioned in the temperature constant zone of the furnace. The temperature of the specimen was controlled with two S-type thermocouples, one at the lower front and one at the top back of the specimen. The displacement was measured with two ceramic rods in tube extensometers and inductive strain sensors outside the furnace. During heating a small preload was used to keep the load line in position. After reaching the test temperature the system required about half an hour to reach thermal equilibrium, at which point the load was applied. Two creep tests per material state were carried out at 750 °C and 250 MPa until rupture. The purpose was to compare the performance of these novel modified TNM alloys subjected to the IHT processing route to the conventional forging route.

A tensile test was also conducted with an INSTRON 3369 machine at room temperature, on an TNM-0.8B-HIP flat sample with an initial gauge length of 70 mm and an initial width of 15 mm, to understand the material behaviour at low temperatures.

### 2.4. Microstructural Evaluation

For the microstructure analysis and crack evaluation, scanning electron microscopy was performed on a section of the polished samples with a FEI NOVA NANOSEM 450 in Backscattered Electrons (BSE) and high-contrast mode. Two zones were analysed, one close to the fracture and the other a few millimetres from the fracture. The former was established at about a millimetre from the fracture to avoid test artefacts, while the latter was a few millimetres from the fracture, to provide greater phase-contrast.

The SEM BSE images were analysed using a procedure similar to the intercept method of ASTM E112, described in reference [23]. Porosity analysis was also carried out with Leica Application Suite (LAS) using the image analysis module. The region of analysis was about 4 mm^2^ size and corresponded to the area close to the fracture surface. For this purpose, high-resolution images were taken with the navigation montage addon in SEM BSE mode, which is capable of measuring cavities of sizes larger than 10 µm in diameter.

## 3. Results

### 3.1. Creep Test

**Figure 6:**
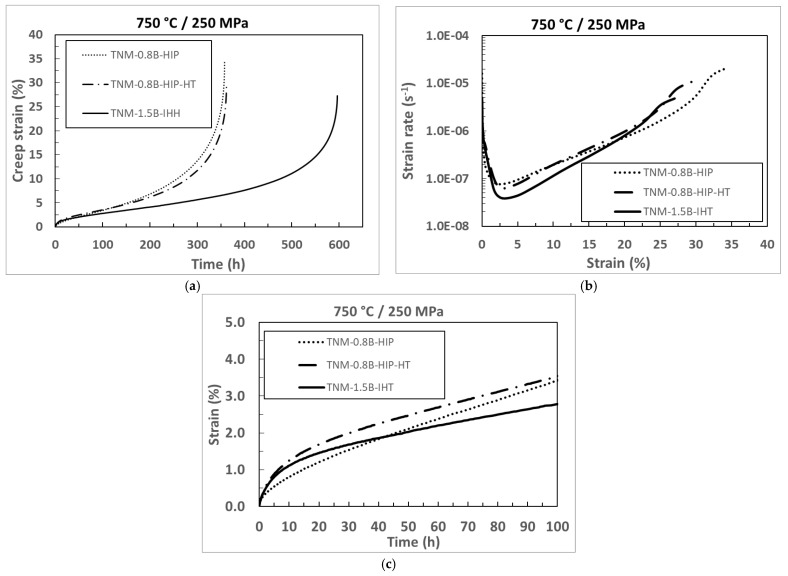
Creep results of the three manufactured samples; (**a**) Creep strain versus time; (**b**) Strain rate versus strain; and (**c**) Creep strain versus time up to 100 h, (detailed from figure (**a**)).

The creep behavior of the three individual material states is compared in Figure 6. Only one test of each material state is shown, as all present similar behaviour. In the strain vs. time plot (Figure 6a), it can be observed that the three curves are overlapping at the beginning, in the primary creep regime. From about 2% strain on, the TNM-1.5-IHT deviates from the other materials and shows a slower accumulation of creep strain. The rupture time of 574 h is more than 1.5 times higher than the two other materials (357 h). This is also represented in the strain rate vs. strain plot, where the TNM-1.5-IHT material reports the lowest minimum creep rate ε_min_ of 4.8 × 10^−8^ s^−1^, (Figure 6b). After reaching the minimum creep rate, all materials display an increase in creep rate until the end of the test. Figure 6c highlights the primary creep regime, where the deviation of the TNM-1.5-IHT material is clearly visible.

### 3.2. Microstructure Evolution during Creep

**Figure 7:**
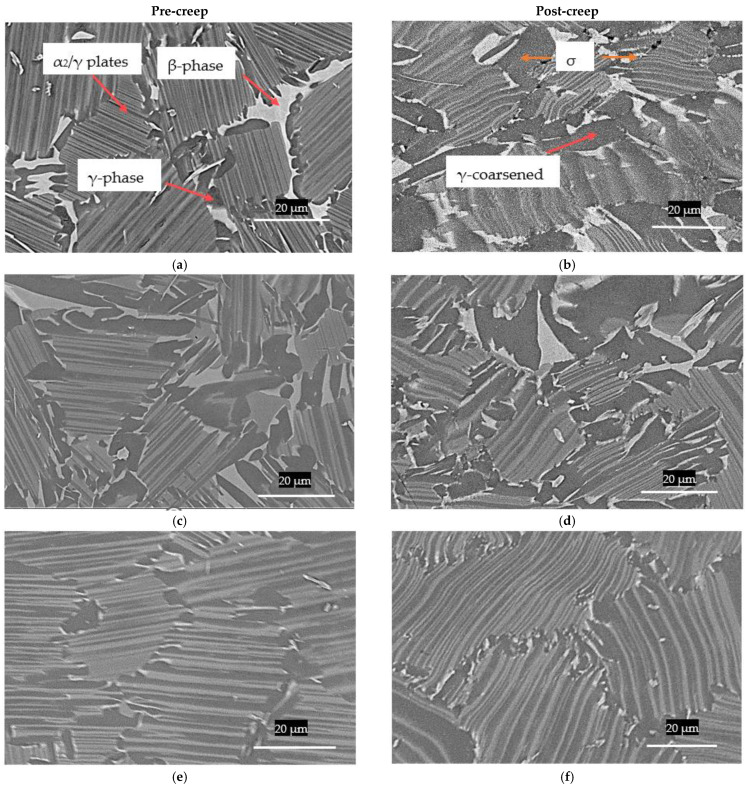
SEM; (**a**) TNM-0.8B-HIP pre-creep, (**b**) TNM-0.8B-HIP post-creep, (**c**) TNM-0.8B-HIP-HT pre-creep, (**d**) TNM-0.8B-HIP-HT post-creep, (**e**) TNM-1.5B-IHT pre-creep, (**f**) TNM-1.5B-IHT post-creep.

Prior to the creep test, both TNM-0.8B-HIP and TNM-0.8B-HIP-HT samples presented a nearly fully lamellar microstructure, with some globular γ-phase and β_0_-phase decorating the colony boundaries. However, the content of β_0_-phase was higher in the former with values of 7.6%, and of 3.9% for the latter. In contrast, the amount of globular γ-phase seemed to be higher in the second material (Figure 7c). Furthermore, the TNM-0.8B-HIP-HT had a lamellar colony size and lamellar spacing smaller than the TNM-0.8B-HIP (Table 3). The TNM-1.5B-IHT on the other hand, displayed a XD lamellar (XDL) microstructure, which is a complex form of fully lamellar (FL) microstructure, with a 0.3% of β_0_-phase (Figure 7e).

After the creep test, there was an increase in the β_0_-phase, with values of 10.4%, 6.9%, and 0.5% for the TNM-0.8B-HIP, TNM-0.8B-HIP-HT, and TNM-1.5B-IHT, respectively. The lamellar spacing also slightly increased during creep (Figure 7), around 0.1 points for TNM-0.8B-HIP and TNM-1.5B-IHT, and approximately 0.2 points for TNM-0.8B-HIP-HT. The lamellar colony size did not display significant changes (Table 3). Of note is the fact that the IHT process produced thinner lamellar spacing due to faster cooling rate in comparison with the slow cooling rate of conventional HIP. Overall, TNM-1.5B-IHT achieved the least variation of microstructural parameters during creep. It also presented a better microstructure for structural applications (Figure 7f), resulting in enhanced creep resistance.

In terms of microstructural evolution during the creep test, the lamellar structure started to decompose due to DRX during the secondary creep, as observed by [1,28]. Figure 7 shows the microstructure before and after the creep test of the three specimens. The non-heat-treated samples (Figure 7b) show that the lamellae colony boundaries decomposed to form newly precipitated coarsened γ and β_0_-phases from the metastable α_2_-phase. There is less evidence of this in heat-treated samples, however (Figure 7d,f).

**Figure 8:**
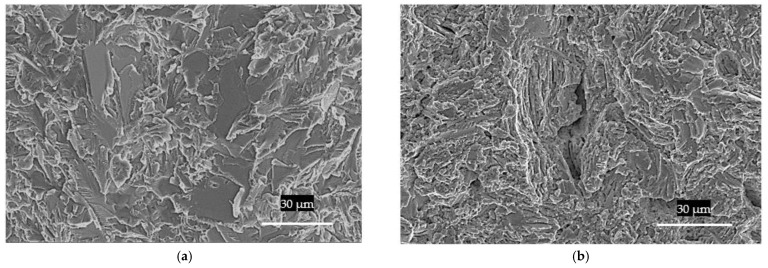
SEM; (**a**) TNM-0.8B-HIP tensile test fracture at room temperature, (**b**) TNM-0.8B-HIP post-creep fracture (750 °C, 250 MPa).

As regards the type of failure for the TNM-0.8B-HIP, Figure 8a illustrates that at room temperature the fracture surface exhibited brittle behaviour, with a sharp fracture surface that appears to emerge from intragranular cracking with no plastic deformation. The failure during the creep test at 750 °C displayed a wave-shaped surface, which appears to be related to plastic deformation (Figure 8b). This indicates that the sample was tested at a temperature above the Brittle-Ductile Transition during creep (BDTC), which is expected to be around 750 °C to 800 °C, as stated by [22]. This plastic behaviour can also be verified in Figure 7b,d,f, in which the lamellar plates wobbled due to plastic deformation.

**Figure 9:**
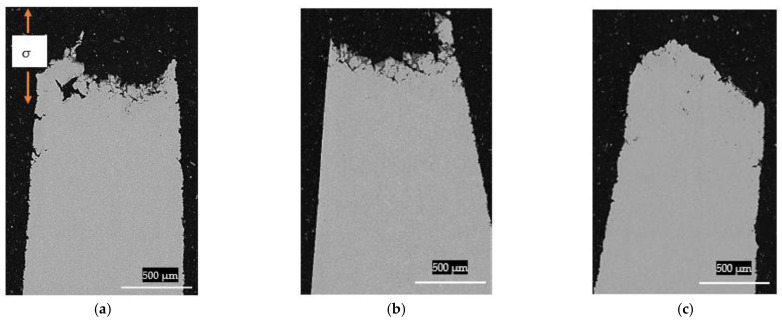
SEM post-creep; (**a**) TNM-0.8B-HIP, (**b**) TNM-0.8B-HIP-HT, (**c**) TNM-1.5B-IHT.

Turning to the porosity generated during the creep test, no major differences were measured (Figure 9). This was despite the fact that the TNM-0.8B-HIP sample had a higher β_0_-phase content and therefore higher plasticity than the other two samples. The measured porosity values for the TNM-0.8B-HIP, TNM-0.8B-HIP-HT and TNM-1.5B-IHT were 0.22%, 0.12%, and 0.13%, respectively.

## 4. Discussion

The main goal of this study is to establish an advanced manufacturing route for TiAl alloys, by integrating HIP and HT into a single step, so-called IHT. For this purpose, the process parameters and workflow were adjusted to develop the required microstructure detailed in references [23,25]. Then, the developed material was creep tested to validate its performance.

Overall, alloy TNM-1.5B-IHT presents superior creep strength compared to the other studied TNM alloys, thanks to its least variation of microstructural parameters during creep. To meet this goal, a microstructure with lamellar colonies in the range of 50–100 μm, nanometre-spaced lamellae, and a low volume fraction of globular γ-grains and no globular β_0_-grains, was developed. From [23,25] previous studies, this superior performance is derived from the adjustment of the chemical composition, the optimization of the solidification cooling rate of the casting process and the application of the integrated HIP heat treatment IHT developed in this work.

### Comparison between Conventional Forging Route and Cast/IHT Process

**Figure 10:**
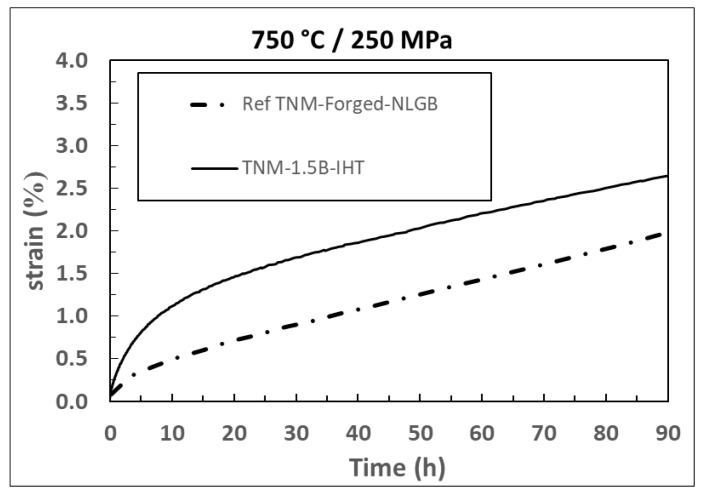
Creep curves at 750 °C and 250 MPa of both novel modified TNM IHT and conventional TNM-forged, after [6].

Figure 10 shows the creep test of both the novel modified TNM-1.5B-IHT and the reference conventional TNM-Forged-NLGB from [6], depending on the processing route of conventional TNM-Forged alloy its creep strength vary, for the sake of simplicity the conventional TNM-Forged-NLGB is set as the reference. These tests were conducted at 750 °C and an initial stress of 250 MPa. It can be observed that the TNM-Forged-NLGB had a lower strain during primary creep than the TNM-1.5B-IHT. However, during secondary creep in which the ε_min_ describes the behaviour of this region, both alloys displayed a similar minimum creep rate, with values of 4.8 × 10^−8^ s^−1^ for the TNM-1.5B-IHT, and 4.9 × 10^−8^ s^−1^ for the TNM-Forged-NLGB.

These results indicate that the cast modified TNM-1.5B-IHT has achieved creep strength similar to the conventionally forged TNM-Forged-NLGB. Although the sample presented a larger colony size and thicker lamellar spacing (Table 4), the colonies were nonetheless well distributed. This good creep behaviour may arise from the lower presence of the soft β-phase and the XDL microstructure. Resulting in a good distribution of the orientations of the lamellae, as the α_2_-phase offers higher stiffness despite having thicker lamellar spacing.

## 5. Conclusions

The aim of this investigation was to develop an advanced thermomechanical processing route for modified TNM alloys, by integrating HIP and HT into a single IHT process. The results show that the modified TNM-1.5B-IHT manufactured by the IHT route presents some advantages over conventional thermomechanical processing of forged TNM alloys, and the following conclusions can be drawn:

- Creep tests reveal that the novel modified TNM-1.5B-IHT alloy exhibits a slower accumulation of creep strain, a lower minimum creep rate ε_min_, 4.8 × 10^−8^ s^−1^, and a rupture time that exceeds that of the other tested materials by a factor of 1.5.
- TNM-1.5B-IHT presents a XDL microstructure composed of an average lamellar colony size of 55 µm and an average lamellar spacing of 0.6 µm, while the amount of β_0_-phase dropped to values below 0.5%. This in conjunction with reduced porosity accounts for the good resistance of this microstructure during creep.
- The modified TNM-1.5B-IHT demonstrates the potential of the effect of chemical composition and casting/IHT manufacturing route to reduce processing time and costs in comparison to conventional forged processes, while achieving similar creep behaviour.

## Figures and Tables

**Table 1:** Nominal chemical composition in at.% of the modified TNM alloys.

Alloy	Ti	Al	Nb	Mo	B
TNM-0.8B	Balanced	43.5	3.5	1	0.8
TNM-1.5B	Balanced	42.5	3.5	1	1.5
TNM-Forged-NLGB	Balanced	43.7	4.1	1	0.1

**Table 2:** Manufacturing and processing routes of the creep tested samples.

Sample	Casting Route	HIP	HT
TNM-0.8B-HIP	PAM + ISM	200 MPa Ar, 1270 °C, 4 h, slow cooling	None
TNM-0.8B-HIP-HT	PAM + ISM	200 MPa Ar, 1270 °C, 4 h, slow cooling	1260 °C, 1 h, 20 MPa Ar, cooling at 30 K/min
TNM-1.5B-IHT	VAR + ISM	Integrated: 1250 °C, 3h, 200 MPa Ar and 1260 °C, 1 h, 200 MPa Ar, 30 K/min	

**Table 3:** Results of the remaining % area of β_0_-phase, lamellar colony size, and α_2_/γ-lamellar spacing of each sample and state.

Sample	State	% Area of β_0_-Phase	Colony Size [µm]	Lamellar Spacing [µm]	ε_min_ [s^−1^]	Time to Creep Rupture [h]
TNM-0.8B-HIP	Pre-creep	7.6	56	0.8	-	-
	Post-creep	10.4	53	0.9	7.8 × 10^−8^	357
TNM-0.8B-HIP-HT	Pre-creep	3.9	51	0.6	-	-
	Post-creep	6.9	65	0.8	6.3 × 10^−8^	362
TNM-1.5B-IHT	Pre-creep	0.3	58	0.6	-	-
	Post-creep	0.5	51	0.7	4.8 × 10^−8^	574

**Table 4:** Comparison of microstructural and mechanical properties of both novel modified TNM IHT and conventional TNM-Forged.

Sample	% Area of β_0_-Phase	Colony Size [µm]	Lamellar Spacing [µm]	ε_min_ [s^−1^]
TNM-1.5B-IHT	0.5	51	0.7	4.8 × 10^−8^
TNM-Forged-NLGB	1	15	0.02	4.9 × 10^−8^
